# Screening of postoperative cerebral hyperperfusion syndrome in moyamoya disease: a three-dimensional pulsed arterial-spin labeling magnetic resonance imaging approach

**DOI:** 10.3389/fnins.2023.1274038

**Published:** 2023-10-19

**Authors:** Feng Gao, Jianhua Cong, Yu Duan, Wei Zhao, Zhenfang Zhu, Yu Zheng, Liang Jin, Ming Ji, Ming Li

**Affiliations:** ^1^Department of Radiology, Huadong Hospital Fudan University, Shanghai, China; ^2^Department of Medical Centre, Huadong Hospital Fudan University, Shanghai, China; ^3^Department of Neurosurgery, Huadong Hospital Fudan University, Shanghai, China; ^4^Department of Radiology, Second Xiangya Hospital, Central South University, Changsha, China; ^5^Department of Magnetic Resonance, Lanzhou University Second Hospital, Lanzhou, China

**Keywords:** moyamoya disease, arterial spin labeling, magnetic resonance imaging, surgery, phase contrast MRI, cerebral hyperperfusion syndrome

## Abstract

**Introduction:**

Moyamoya disease (MMD) is associated with a risk of postoperative cerebral hyperperfusion syndrome (CHS) after revascularization surgery. This study aimed to explore the feasibility of using three-dimensional pulsed arterial spin labeling (3D PASL) and phase contrast (PC) magnetic resonance imaging (MRI) for predicting CHS occurrence in patients with MMD before revascularization surgery.

**Methods:**

Overall, 191 adult patients (207 hemispheres) with MMD who underwent combined revascularization surgery were included in this study. Preoperative 3D PASL-MRI and PC-MRI were performed before surgery. The PASL-MRI data were analyzed using SPM12. Patient clinical information, average flow, and preoperative cerebral blood flow (CBF) were compared between the non-CHS and CHS groups.

**Results:**

Among the patients, 45 (21.74%) developed CHS after revascularization surgery. No significant differences were noted in age, sex, clinical symptoms, hypertension, diabetes, surgical side, or history of revascularization surgery between the non-CHS and CHS groups. However, the average flow in the superficial temporal artery was significantly lower in the CHS group than in the non-CHS group (*p* < 0.05). Furthermore, 11 clusters of preoperative CBF values were significantly greater in the CHS group than in the non-CHS group [*p* < 0.05, false discovery rate (FDR) corrected]. A significant correlation was also observed between the preoperative time-to-flight MR angiography (MRA) scores and CBF values in patients with MMD (p < 0.05).

**Conclusion:**

Compare patients with lower preoperative CBF and higher preoperative average flow in the STA, patients with higher preoperative CBF and lower preoperative average flow in the STA are more likely to develop postoperative CHS Preoperative PASL-MRI and PC-MRI examinations may help to screen patients at high risk of developing CHS after revascularization surgery.

## 1. Introduction

Moyamoya disease (MMD) is a chronic, occlusive cerebrovascular disease of unknown etiology. It is characterized by steno-occlusive changes in the terminal part of the internal carotid artery and an abnormal development of vascular network (moyamoya vessels) at the base of the brain (Research Committee on the Pathology and Treatment of Spontaneous Occlusion of the Circle of Willis, and Health Labour Sciences Research Grant for Research on Measures for Infractable Diseases, 2012). The symptoms of MMD primarily result from two major causes: brain ischemia (transient ischemic attacks, stroke, and seizures) and the compensatory response to ischemia. These include the common symptom of headache, which may be due to dilated transdural collaterals, and another symptom of hemorrhage, which is caused by fragile moyamoya vessels (Scott and Smith, 2009).

MMD often presents at younger ages, and its progressive characteristics can lead to irreversible brain damage due to prolonged brain hypoperfusion and multiple cerebral infarction/hemorrhagic events, resulting in severe disability and a poor prognosis (Fung et al., 2005). Revascularization surgery, including indirect, direct, and combined procedures, is recommended to improve cerebral perfusion, reduce the incidence of cerebrovascular events, and prevent neurocognitive decline (Zhang et al., 2020). Indirect procedures in combination with direct bypass offer the advantages of both direct and indirect bypass and are recommended for patients with MMD (Esposito et al., 2018). However, it should be noted that the superficial temporal artery-middle cerebral artery (STA-MCA) bypass provides only a limited blood supply (Kazumata et al., 2014).

Patients with MMD are more likely to experience postoperative cerebral hyperperfusion syndrome (CHS), compared to patients with other atherosclerotic diseases (Fujimura et al., 2011). Postoperative CHS can cause transient neurological symptoms, including permanent neurological defects, as intracranial vasculature pressure increases, which may elicit intracranial hemorrhage and even hematogenous cerebral edema (Park et al., 2018). Multiple studies recommend maintaining low perioperative blood pressure to reduce the risk of postoperative CHS (Hayashi et al., 2012). However, lowering blood pressure can also result in cerebral hypoperfusion and an increased risk of cerebral infarction after surgery (Hayashi et al., 2012). Some studies suggest induction of hypertension and hypervolemia during the perioperative period to prevent neurologic complications (Li et al., 2022). The identification of high-risk patients for developing postoperative CHS before surgery is crucial. Implementing appropriate treatment during the perioperative period is essential in preventing postoperative CHS and avoid adverse events at the same time, this may improve the overall prognosis of these patients (Østergaard et al., 2014).

Various imaging modalities can evaluate cerebral perfusion status. Computed tomography and single-photon emission computed tomography are radiation-based techniques, while magnetic resonance imaging (MRI) is a nonradiative alternative. MRI techniques include perfusion imaging, dynamic susceptibility contrast, and arterial spin labeling (ASL). ASL-MRI is magnetically labeled by the radiofrequency (RF) pulse protons of arterial blood flowing into the brain as an endogenous tracer (Zaharchuk et al., 2009). The cerebral blood flow (CBF) of brain tissues is quantified by estimating the labeled arterial blood water (Goetti et al., 2013). Studies have shown that even patients with normal renal function may have chronic gadolinium deposition in brain tissue after repeated intravenous injections of gadolinium-based contrast agents (McDonald et al., 2015). The ASL sequence presents a viable alternative for gadolinium-based contrast perfusion imaging. ASL permits repeated examinations without the risk of contrast leakage or residuals, and its clinical utility spans from ischemic stroke to arteriovenous malformations. ASL also has the potential to measure CBF patterns in patients with MMD (Federau et al., 2017). In addition, quantitative phase-contrast MR imaging (PC-MRI) can measure blood flow in a specified vessel without the need of contrast agent.

Patients with MMD, who are typically young, require regular MR examinations throughout their lifetime. This renders ASL sequences and quantitative PC-MRI highly suitable those patients. This study aimed to identify potential risk factors for postoperative CHS in adult patients with MMD who underwent surgical revascularization using three-dimensional (3D) pulsed arterial spin labeling (PASL)-MRI and PC-MRI were employed. We anticipate that the findings of this study contribute to the advancement of personalized medicine, implementation of appropriate treatment during the perioperative period, and improved patient care in Moyamoya disease.

## 2. Materials and methods

### 2.1. Clinical data and participants

This study was approved by the Ethics Committee of HuaDong Hospital (approval no. 2018030). All examinations in current study are in accordance with relevant regulations and guidelines. All patients in current study provided written informed consent.

**Figure 1:**
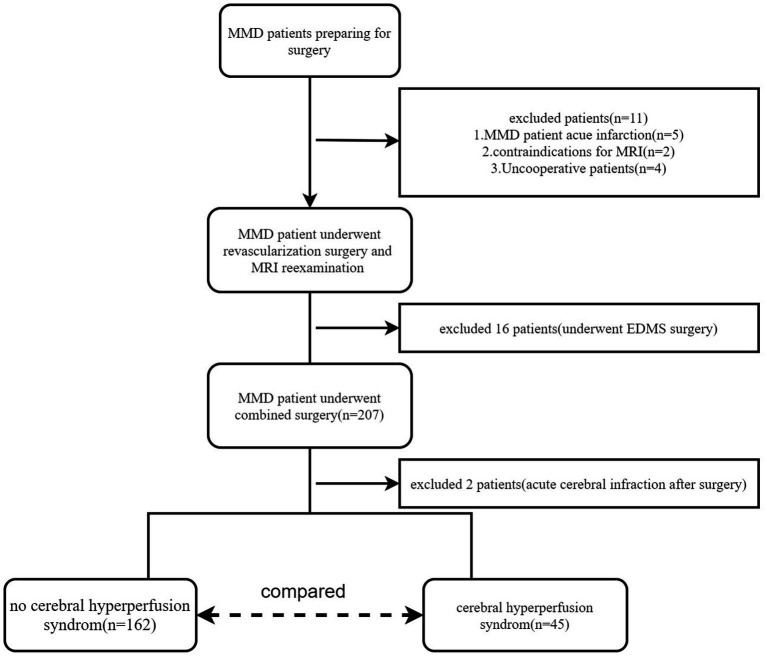
Flowchart describing the patient inclusion process.

From August 2018 to September 2022, a total of 191 adult patients with MMD (mean age, 43.80 ± 11.29 years; range, 18–65 years; female, 102; male, 89) were included in this study. A small subset (16/191) of these participants underwent bilateral revascularization surgery. Finally, 207 hemispheres were included in this study. Digital subtraction angiography (DSA) was used to diagnose MMD (Fukui, 1997). The inclusion criteria were as follows: (i) patients aged **≥**18 years; (ii) those with no contraindication to MR examination; and (iii) those who underwent combined revascularization surgery [encephalo-duro-myo-synangiosis (EDMS) combines STA-MCA bypass]. The exclusion criteria were as follows: (i) patients with any other neurosurgical disease and (ii) those who underwent only STA-MCA bypass or EDMS surgery. The enrolment flowchart is shown in Figure 1. In this study, the preoperative and postoperative blood pressure and blood glucose will be carefully monitored for patients with hypertension and diabetes (blood glucose is controlled within the range of 7–9 mmol/L; systolic blood pressure is controlled within the range of 120–130 mmHg).

### 2.2. MRI examination protocol

All MRI examinations were performed using a 3-T machine (Siemens Prisma; Siemens Medical Solutions) with a 32-channel head–neck coil. Preoperative MRI examinations were conducted within 1 week before surgery.

The parameters for 3D PASL acquisition were as follows: label time: 700 ms; post-labeling delay time: 1,290 ms; inversion time: 1,990 ms; field of view (FOV): 192 × 192; voxel size: 3 × 3 × 3 mm; slice thickness: 3 mm with a 1.5 mm gap; time to echo (TE): 16.18 ms; repetition time (TR): 4,600 ms; axial slices: 40; number of excitations: 4; and time of acquisition (TA): 296 s. The other MRI sequence parameters are listed in Table 1.

**Table 1:** MRI scanning sequences and parameters.

	TR/TE (ms)	Thickness (mm)	Flip angle (°)	Intersection gap	TA	FOV (cm^2^)	Matrix	*b* value
T1WI	230/2.46	5	70	30%	25 s	22 × 22	256 × 192	/
T2WI	5000/117	5	90	30%	54 s	22 × 22	384 × 281	/
FLAIR	8000/85	5	140	20%	1 min 36 s	22 × 22	256 × 162	/
DWI	1300/62	5	192	30%	30 s	24 × 24	192 × 192	0,1,000
T1-MPRAGE	2300/2.32	0.9	8	50%	5 min 22 s	26 × 26	256 × 256	/
TOF-MRA	21/3.43	1	18	−18.75%	5 min 13 s	22 × 22	320 × 180	/
PC-MRI (pre-scan)	84.6/4.86	6	20	20%	19 s	34 × 34	192 × 68	/
PC-MRI	73.08/7.54	4	10	20%	5 min 29 s	18 × 18	336 × 336	/

TR, time of repetition; TE, time of echo; FOV, field of view; TA, time of acquisition; FLAIR, fluid-attenuated inversion-recovery sequence; DWI, diffusion-weighted imaging; T1-MPRAGE, T1 magnetization prepared rapid acquisition gradient echo; TOF-MRA, time-to-flight MR angiography; PC-MRI, phase-contrast MRI.

The 3D-time-of-flight (TOF) MR angiography (MRA) sequence, from the top of the skull to the common carotid artery, was located using the T1- MPRAGE sequence. The TOF-MRA sequence is reconstructed by maximum intensity projection (MIP), and it was used to locate the PC MRI sequence. The detailed procedure for measuring the average flow in the internal carotid artery (ICA), basilar artery (BA), and superficial temporal artery (STA) is consistent with a previous study (Gao et al., 2019).

### 2.3. MRI data Preprocessing

The 3D PASL data were analyzed using SPM12 software ^1^ and the ASL toolbox^2^ (Wang et al., 2008). In this study, CBF was quantitatively assessed at the whole-cerebral voxel level, using an individual’s T1-MPRAGE anatomical scan as a reference. The detailed procedure for ASL image analysis has been previously described (Huang et al., 2022). CBF map values were reported in absolute units (mL/100 g/min).

The MIP images of the MRA sequence were used to assess the MRA scores of the intracranial arteries. Two senior neuroradiologists with more than 10 years of experience evaluated the MRA scores following previously reported criteria (Houkin et al., 2005). The total MRA scores ranged from 0 to 20.

### 2.4. Diagnosis of CHS, grouping, and analysis

In this study, postoperative symptomatic CHS was diagnosed if patients experienced severe headaches, seizures, or new neurological deficits developed after surgery, and brain CT images or/and diffusion magnetic resonance images shows neither definite hematomas nor definite acute infarctions (van Mook et al., 2005; Iwata et al., 2014). The diagnosis of postoperative symptomatic CHS was made by a senior neurosurgeon who was blinded to the study. Based on the presence or absence of CHS, the patients were divided into CHS and non-CHS groups. The preoperative CBF status and average flow in the ICA, STA, and BA were compared between the two groups.

### 2.5. Statistical analyses

An independent t-test was used to compare the age and average flow in the ICA, STA, and BA between the CHS and non-CHS groups. The average flows of the target blood vessel were separately measured for the left and right sides, and the data from both sides were averaged. The Chi-squared test was used to compare categorical variables, such as sex, clinical symptoms, diabetes, hypertension, surgical side, and history of revascularization surgery between the CHS and non-CHS groups. Statistical analyses were conducted using the Statistical Package for the Social Sciences (version 24.0; IBM SPSS) and an automated anatomical labeling atlas (AAL) template (Ashburner, 2007).

The CBF maps were statistically analyzed using second-level statistical procedures implemented in SPM12, based on a generalized linear model. The preoperative CBF in the CHS and non-CHS groups was compared using a two-sample *t*-test. Only CBF of cerebral was selected for analysis, as MMD rarely affects the cerebellum. Sex and age were included as covariates in the regression analysis. The correlation between preoperative CBF and MRA scores was analyzed using multiple regression and regression of the z-transformed correlation coefficients. A statistically significant difference was considered at *p* < 0.05 after false discovery rate (FDR) correction.

The xjview toolbox^3^ was used to visualize CBF clusters. Clusters with significant differences and correlations are displayed in pseudo-colors on a calibrated standard brain map. The voxel sizes of the peak intensities and also their Montreal Neurological Institute coordinates are listed.

## 3. Results

In this study, 45 of 207 patients with MMD (21.74%) developed symptomatic CHS after surgery. The main symptoms of CHS included speech impairment (15 cases), severe headache (12 cases), decreased muscle strength (upper limbs in 3 cases and lower limbs in 5 cases), persistent vomiting (4 cases), dysphagia (3 cases), seizures (2 cases), and face and eye pain (1 case). These symptoms typically appeared within 1–9 days (3.87 ± 1.87 days) after surgery and improved before the patients were discharged from our hospital.

**Figure 2:**
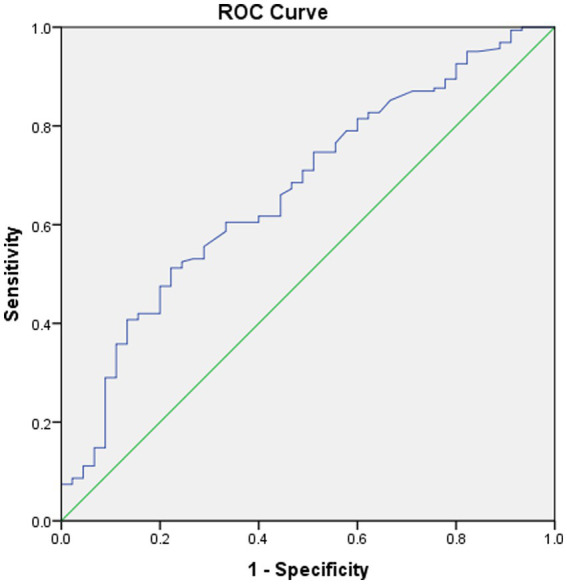
Received operating characteristic (ROC) curve of the average flow of STA in predicting postoperative CHS. The area under the curve (AUC) is 0.67.

**Figure 3:**
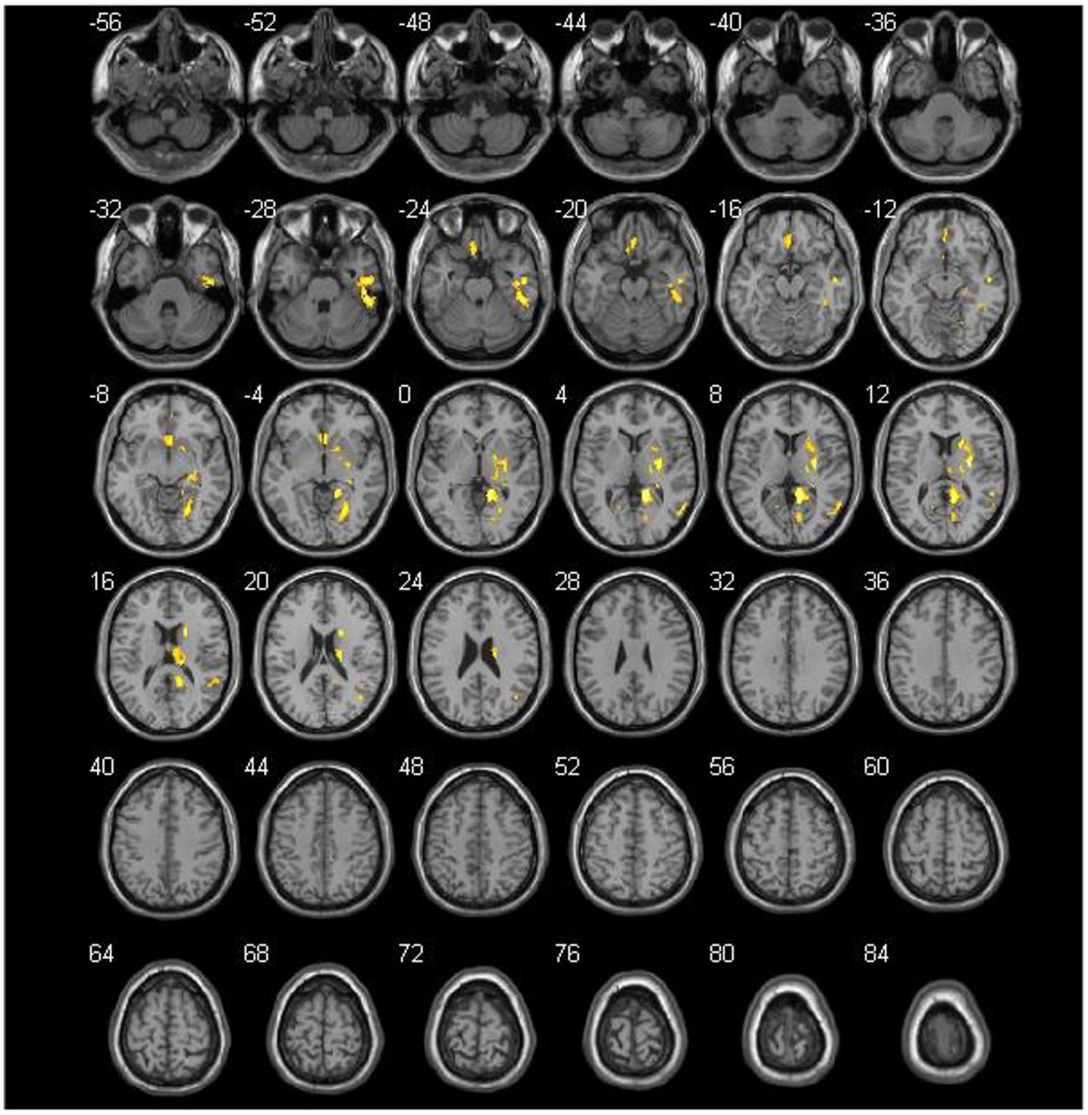
Comparison of the preoperative cerebral blood flow between cerebral hyperperfusion syndrome (CHS) and non-CHS groups. Significant regions are (false discovery rate corrected *p* < 0.05) illustrated in warm colors for increased values. Significant differences are revealed in the following brain regions: right inferior temporal gyrus (Temporal_Inf_R), left gyrus rectus (Rectus_L), right lenticular nucleus, putamen (Putamen_R), right olfactory cortex (Olfactory_R), right precuneus (Precumeus_R), right hippocampus (Hippocampus_R), right middle temporal gyrus (Temporal_Mid_R), left calcarine fissure and surrounding cortex (Calcarine_L), right superior temporal gyrus (Temporal_Sup_R).

No significant differences were noted in age, sex, clinical symptoms, hypertension, diabetes, surgical side, or history of revascularization surgery between the two groups (Table 2). The average preoperative flows in the ICA and BA were not significantly different between the two groups (Table 2). However, before surgery, the average STA flow was significantly lower in the CHS group than in the non-CHS group (*p* < 0.05; Table 2). The ROC curve of the average flow of STA in predicting postoperative CHS is shown in Figure 2, and the area under curve (AUC) was 0.67. The two-sample t-test showed the significant differences in preoperative CBF status between the two groups. Specifically, 11 clusters of preoperative CBF were significantly higher in the CHS group than in the non-CHS group. These clusters were mainly located in the following brain areas: right inferior temporal gyrus (Temporal_Inf_R), left gyrus rectus (Rectus_L), right lenticular nucleus, putamen (Putamen_R), right olfactory cortex (Olfactory_R), right precuneus (Precumeus_R), right hippocampus (Hippocampus_R), right middle temporal gyrus (Temporal_Mid_R), left calcarine fissure and surrounding cortex (Calcarine_L), right superior temporal gyrus (Temporal_Sup_R) (Table 3; Figure 3).

**Table 2:** Clinical information of MMD patients (207 brain hemispheres).

	CHS	Non-CHS		*p*
No. of patients (%)	45 (21.74%)	162 (78.26%)		
Age	40.47 ± 13.82	44.04 ± 10.59	*t* = −1.61	0.113
Gender			*χ*^2^ = 0.281	0.596
Male	22	72		
Female	23	90		
Hypertension	29	89	*χ*^2^ = 1.299	0.254
Diabetes	21	72	*χ*^2^ = 0.385	0.535
Clinical symptom			*χ*^2^ = 0.443	0.801
Ischemia	26	85		
Hemorrhage	15	59		
Nonspecific	4	18		
Surgery side			*χ*^2^ = 0.238	0.626
Left	24	93		
Right	21	69		
Revascularization surgery history			*χ*^2^ = 2.748	0.097
No	45	114		
Yes	10	48		
Average flow (mL/s)				
Superficial temporal artery	0.24 ± 0.19	0.37 ± 0.29	*t* = −3.552	0.010
Internal carotid artery	2.17 ± 1.69	2.37 ± 1.92	*t* = −0.657	0.512
Basilar artery	1.28 ± 1.17	1.57 ± 1.33	*t* = −1.312	0.191

CHS, cerebral hyperperfusion syndrome; non-CHS, non-cerebral hyperperfusion syndrome.

**Table 3:** Comparison of the preoperative cerebral blood flow between CHS and non-CHS groups.

Cluster	Brain region	Cluster size	Peak MNI coordinates(x, y, z)	Peak intensity	T value	*p*_FDR-corr_
1	Temporal_Inf_R	518	54, –38, –28	6.176	6.18	0.000
2	Rectus_L	167	−6, 20, –26	4.398	4.40	0.005
3	Putamen_R	42	16, 8, –8	3.999	4.00	0.011
4	Olfactory_R	123	2, 16, –6	4.282	4.28	0.006
5	Precumeus_R	691	12, –46, 6	5.745	5.74	0.000
6	Hippocampus_R	81	32, –30, –4	4.321	4.32	0.006
7	Putamen_R	638	28, –12, 12	5.034	5.03	0.001
8	Temporal_Mid_R	71	58, –60, 6	4.525	4.53	0.004
9	Calcarine_L	28	−14, −60, 10	3.496	3.50	0.028
10	Temporal_Sup_R	51	56, –46, 14	3.710	3.71	0.019
11	Temporal_Mid_R	26	44, –66, 22	4.059	4.06	0.010

CHS, cerebral hyperperfusion syndrome; non-CHS, non-cerebral hyperperfusion syndrome.

**Figure 4:**
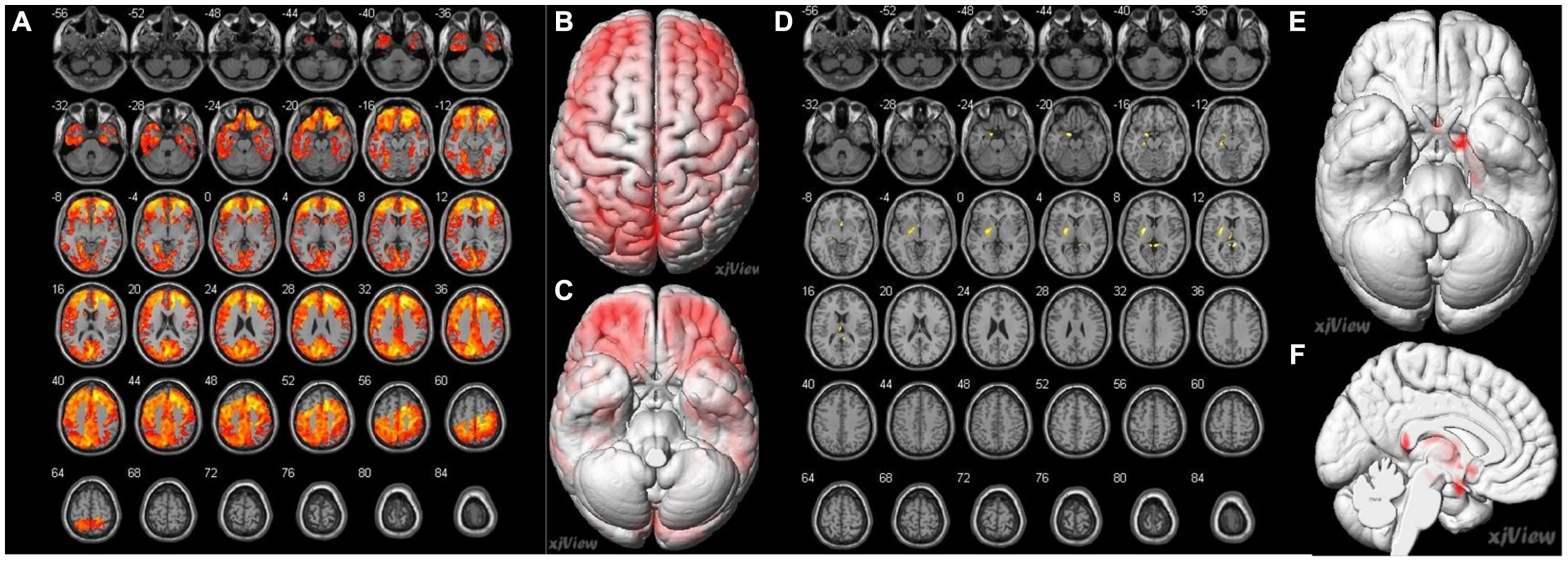
Correlation of preoperative cerebral blood flow (CBF) between brain regions and preoperative MRA sores in patients with MMD. **(A–C)** A negative correlation (false discovery rate corrected p < 0.05) is identified between MRA scores and the following preoperative brain area: bilateral superior temporal gyrus (Temporal_Sup_R, L), right superior frontal gyrus, medial (Frontal_Sup_Medial_R), right middle temporal gyrus (Temporal_Mid_R), right inferior occipital gyrus (Occipital_Inf_R), right middle occipital gyrus (Occipital_Mid_R), right superior parietal gyrus (Parietal_Sup_R). **(D–F)** Positive correlations (false discovery rate corrected p < 0.05) were identified between the MRA scores and the following preoperative brain areas: the bilateral posterior cingulate gyrus (Cingulum_Post_R, L), left parahippocampal gyrus (ParaHippocampal_L), left hippocampus (Hippocampal_L), left olfactory cortex (Olfactory_L), left lenticular nucleus, putamen (Putamen_L), and left thalamus (Thalamus_L).

Spearman’s correlation coefficients were calculated to evaluate the relationship between the preoperative CBF values and MRA scores of intracranial arteries in patients with MMD. The results revealed a significant correlation between preoperative MRA scores and CBF values in patients with MMD (*p* < 0.05). The brain areas related to MRA scores in the CHS group are shown in Table 4 and Figure 4. Negative correlations were observed between MRA scores and the following preoperative brain areas: the bilateral superior temporal gyri (Temporal_Sup_R,L), right superior frontal gyri, medial (Frontal_Sup_Medial_R), right middle temporal gyri (Temporal_Mid_R), right inferior occipital gyri (Occipital_Inf_R), right middle occipital gyri (Occipital_Mid_R), and right superior parietal gyri (Parietal_Sup_R). On the other hand, positive correlations were identified between the MRA scores and the following preoperative brain areas: the bilateral posterior cingulate gyrus (Cingulum_Post_R,L), left parahippocampal gyrus (ParaHippocampal_L), left hippocampus (Hippocampal_L), left olfactory cortex (Olfactory_L), left lenticular nucleus, putamen (Putamen_L), and left thalamus (Thalamus_L).

**Table 4:** Correlation between the preoperative CBF values and MRA scores of intracranial arteries in the CHS group.

Cluster	Brain region	Cluster size	Peak MNI coordinates(x, y, z)	Peak intensity	T value	*p*_FDR-corr_
Negative						
1	Frontal_Sup_Medial_R	81,668	12, 44, 32	10.291	10.29	0.000
2	Temporal_Mid_R	504	70, –34, 0	4.499	3.68	0.001
3	Occipital_Inf_R	174	36, –88, –12	3.542	3.54	0.001
4	Occipital_Mid_R	33	36, –96, 8	3.420	3.42	0.001
5	Temporal_Sup_R	64	50, –30, 6	2.375	2.37	0.019
6	Temporal_Sup_L	147	−44, –36, 14	3.056	3.06	0.003
7	Parietal_Sup_R	39	36, –74, 50	2.962	2.96	0.004
Positive						
1	ParaHippocampal_L	107	−16, 2, –18	−5.948	5.95	0.000
2	Hippocampal_L	50	−22, –20, –12	−4.602	4.60	0.002
3	Olfactory_L	34	0, 6, –8	−5.660	5.66	0.000
4	Putamen_L	345	−22, –6, 10	−6.202	6.20	0.000
5	Cingulum_Post_L	27	−4, –38, 10	−5.689	5.69	0.000
6	Cingulum_Post_R	85	4, –38, 12	−6.687	6.69	0.000
7	Thalamus_L	40	−2, –18, 16	−5.204	5.20	0.000

CHS, cerebral hyperperfusion syndrome; non-CHS, non-cerebral hyperperfusion syndrome; MRA, time-to-flight MR angiography.

## 4. Discussion

In this study, 3D PASL sequences were used to evaluate cerebral perfusion in patients with MMD. The results indicate that the preoperative 3D PASL-CBF in certain brain areas was significantly higher in the CHS group than in the non-CHS group. There was a significant correlation between the preoperative MRA and CBF values in patients with MMD. The utilization of 3D PASL sequences and PC MRI may help to identify patients with MMD who are at a higher risk of accruing postoperative symptomatic CHS.

The development of new pathological vessels in MMD is caused by chronic brain hypoperfusion and ischemia. These new compensatory vessels are pathological vessels. The cerebrovascular reactivity and autonomic regulation are impaired. Consequently, after successful revascularization surgery, these vessels are unable to control the increased cerebral blood flow, leading to the occurrence of symptomatic CHS (Hayashi et al., 2012). In this study, the incidence of postoperative CHS was found to be 21.74% (45/207), which is consistent with previous studies (Fujimura et al., 2011, 2012). The reduced cerebral metabolism and downregulation of cortical neurotransmitter receptor function by CHS can lead to cerebral function impaired (Shimada et al., 2018). During the perioperative period, it is crucial to take reasonable preventive measures for high-risk patients who may come up with CHS after surgery.

Further, no significant differences were observed in age, sex, clinical symptoms, hypertension, diabetes, surgical side, or history of revascularization surgery between the two groups. Although symptoms related to ischemia are the most common clinical manifestation of MMD, 10.63% (22/207) of the patients were asymptomatic which is consistent with previous studies, that 1.5% ~ 17.8% of patients with MMD are asymptomatic (Baba et al., 2008). Current study showed that hemorrhage-onset symptoms were observed in approximately 35.75% (74/207) of the patients. Among patients with postoperative CHS, 57.78% (26/45) and 33.33% (15/45) presented with ischemic- and hemorrhage-onset symptoms, respectively. Whether patients with hemorrhagic- or ischemia-onset symptoms more often come up with postoperative CHS remains controversial (Fujimura et al., 2009; Hayashi et al., 2010). Different study populations, preoperative management, and surgical procedures may have contributed to this discrepancy.

Previous studies have demonstrated that symptomatic CHS can be diagnosed based on specific postoperative clinical symptoms (van Mook et al., 2005; Iwata et al., 2014). In the present study, the most prevalent clinical symptoms of CHS were headache and speech impairment, which was consistent with previous study (Egashira et al., 2017).

PC MRI was used to calculate preoperative blood flow in the ICA, BA, and STA. The target vessels for bypass surgery were MCA (branch of ICA) and STA (branch of the external carotid artery). This study showed that the average flow in the ICA and BA had not significantly different between the two groups. However, the preoperative average STA flow was significantly lower in the CHS than in the non-CHS group (*p* < 0.05). Cerebral reconstruction surgery artificially lowers blood pressure and increases blood flow in the distal STA. Patients with lower vessel flow before surgery may experience a more substantial increase in blood flow volume after surgery. The average STA flow increases significantly after cerebrovascular reconstruction surgery, which greatly affects the blood supply to the brain tissue. This may be the reason why patients with a lower average STA flow before surgery were more likely to develop symptomatic CHS after reconstruction surgery.

The preoperative CBF status measured by PASL was compared between the non-CHS and CHS groups. The importance of ASL sequence has been well-validated in studies on ischemic brain diseases (Soman et al., 2020). In patients with MMD, extensive collateral circulation may cause vascular territory boundaries to shift. In this study, CBF was assessed quantitatively based on the whole-cerebral voxel level but not on a specific cerebral artery-supplied brain region. This method of measurement can aid in direct analysis of the original data, while simultaneously avoiding the effects of malacia or chronic hemorrhagic foci.

The results of the current study indicate that preoperative 3D PASL-CBF was higher in the CHS group than in the non-CHS group in some brain areas of the frontal lobe, temporal lobe, limbic lobe, lentiform nucleus, and white matter, which are typically supplied by the ICA and MCA (Table 3; Figure 3). The PCA is rarely involved in MMD. In this study, 29.95% (62/207) of the patients showed PCA involvement (27 cases with unilateral and 35 cases with bilateral PCA involvement). The ischemic status of MMD is different from other internal carotid artery stenosis caused by atherosclerotic disease. In MMD, as the recipient’s vessels are relatively small, postoperative CHS is not only caused by an increase in postoperative bypass flow but may also be caused by vasodilation due to long-term chronic ischemia (Chen and Tu, 2022). In MMD, stenosis of the ICA and its terminal branches may lead to the development of moyamoya vessels and compensatory vasodilation, which may extend the time to peak and mean transmission time, while CBF is increased as a compensatory mechanism. In this study, preoperative CBF was also higher in the CHS group than in the non-CHS group, indicating that the collateral circulation status before surgery was slightly better in the CHS group.

Impaired cerebral autoregulation is related to postoperative cerebral hyperperfusion, which is mediated by endothelial dysfunction. After surgery, cerebral vascular reactivity cannot respond appropriately to the increased blood flow as it is impaired and requires several days to adapt to the new state (Bernstein et al., 1984). A new vascular pathway was established after revascularization surgery, which may have a high priority. This means the blood flow pattern was reversed after revascularization surgery, led to hyperperfusion in the operation area and manifested as hypoperfusion remote from the site of anastomosis (Bao et al., 2022). The clinical symptoms improved after several days of the revascularization surgery.

Our study showed a negative correlation between preoperative MRA scores and CBF values in some brain regions, especially in the temporal and frontal lobes (*p* < 0.05; Table 4; Figure 4). This observation is consistent with previous ASL reports stating that with the severity of intracranial arterial steno-occlusions, the CBF value decreases (Noguchi et al., 2013). Our study also showed that a small part of the brain region exhibited a positive correlation between preoperative MRA scores and CBF values, especially in the limbic lobe of the left cerebral cortex (*p* < 0.05, Table 4; Figure 4). A previous study showed that the dominant language functional area is the left hemisphere and the functional areas are sensitive to CBF changes; therefore, it required higher CBF stability (Lu et al., 2019). In addition, impaired cerebrovascular reactivity is more evident in the left hemisphere (Mukerji et al., 2015). Therefore, when the blood supply to the brain is insufficient, moyamoya vessels may preferentially supply blood to the dominant brain areas. However, as the sample size in this study was limited, this result requires further verification.

In the current study, non-invasive 3D PASL sequences and PC-MRI were used to access cerebral perfusion and blood flow in patients with MMD. The results indicate that the sequences may help to identify patients with MMD at a higher risk of developing postoperative symptomatic CHS. CBF in this study was assessed quantitatively based on the whole-cerebral voxel level, as this method avoids the effects of malacia or chronic hemorrhagic foci, which are often seen in patients with MMD. This measurement method also increases the reliability of this study.

Our study has few limitations. First, it did not include a comparison with other traditional methods for measuring cerebral perfusion status. Previous studies have compared the ability of ASL-MRI to assess hemodynamic status using nuclear medicine methods (Yun et al., 2016). The results of ASL perfusion MRI can be influenced by moyamoya vessels, which may lead to an underestimation of CBF values (Zaharchuk et al., 2011). However, recent studies have suggested that the overestimation effects on the calculated CBF using single post-label delay ASL can be negligible in patients with MMD (Fahlström et al., 2020). A validation study that compares it with other methods and re-evaluates it will be conducted in the future. Second, symptomatic CHS was diagnosed mainly based on the clinical features of patients (van Mook et al., 2005). However, these clinical features are not unique to patients with postoperative CHS. Furthermore, a comparison of postoperative CBF with preoperative CBF status will be conducted in future studies to further investigate changes in CBF. In this study we considered the possible influence of diabetes and hypertension for the results, but education and medication may affect the results. The data of this study was small, especially the CHS group. We will increase the number of cases in future study to further validate the results.

To further enhance our understanding and improve clinical practice, future research should focus on conducting comparative analyses between ASL-MRI and traditional methods, exploring the impact of collateral moyamoya vessels on ASL perfusion MRI results, refining diagnostic criteria for postoperative symptomatic CHS, and evaluating changes in cerebral perfusion by comparing postoperative CBF with preoperative CBF status. Addressing these aspects can contribute to advancing our knowledge and application of ASL-MRI in assessing cerebral perfusion and optimizing patient management in moyamoya disease.

## 5. Conclusion

Patients with higher preoperative PASL-CBF and lower preoperative average STA flow were more likely to develop postoperative symptomatic CHS, compared with those with lower preoperative PASL-CBF and higher preoperative average STA flow. Preoperative 3D PASL sequencing and PC MRI may assist in identifying patients at high risk of developing postoperative CHS before surgery and in determining the most suitable treatment strategy during the perioperative period.

## Data availability statement

The raw data supporting the conclusions of this article will be made available by the authors, without undue reservation.

## Ethics statement

The studies involving humans were approved by The ethic committee of Huadong hospital. The studies were conducted in accordance with the local legislation and institutional requirements. Written informed consent for participation in this study was provided by the participants’ legal guardians/next of kin. Written informed consent was obtained from the individual(s) for the publication of any potentially identifiable images or data included in this article.

## Author contributions

FG: Conceptualization, Investigation, Methodology, Project administration, Software, Supervision, Visualization, Writing – original draft. YD: Validation, Visualization, Writing – original draft, Formal analysis, Investigation, Methodology, Resources. WZ: Conceptualization, Formal analysis, Investigation, Methodology, Project administration, Resources, Supervision, Validation, Visualization, Writing – review & editing. ZZ: Data curation, Formal analysis, Investigation, Methodology, Visualization, Writing – review & editing. YZ: Data curation, Investigation, Methodology, Software, Validation, Visualization, Writing – original draft. LJ: Conceptualization, Funding acquisition, Investigation, Methodology, Project administration, Resources, Writing – review & editing. MJ: Investigation, Methodology, Validation, Visualization, Writing – review & editing. ML: Funding acquisition, Investigation, Methodology, Project administration, Resources, Supervision, Validation, Visualization, Writing – review & editing. JC: Investigation, Software, Validation, Writing – review & editing.

## Footnotes

^1^http://www.fil.ion.ucl.ac.uk/spm

^2^http://cfn.upenn.edu/zewang/ASLtbs.php

^3^http://www.Alivelearn.p/xjview

## References

[ref1] Ashburner J. (2007). A fast diffeomorphic image registration algorithm. NeuroImage 38, 95–113. doi: 10.1016/j.neuroimage.2007.07.007 17761438

[ref2] Baba T. Houkin K. Kuroda S. (2008). Novel epidemiological features of Moyamoya disease. J. Neurol. Neurosurg. Psychiatry 79, 900–904. doi: 10.1136/jnnp.2007.130666, PMID: 18077479

[ref3] Bao Y. Yu F. Wei L. Zhu W. Wang L. Ding H.. (2022). Association between cognitive decline and altered cerebral perfusion in adults with Moyamoya disease after revascularization. Cerebrovasc. Dis. 51, 764–773. doi: 10.1159/000524240, PMID: 35477140

[ref4] Bernstein M. Fleming J. F. Deck J. H. (1984). Cerebral hyperperfusion after carotid endarterectomy: a cause of cerebral hemorrhage. Neurosurgery 15, 50–56. doi: 10.1227/00006123-198407000-00010 6472594

[ref5] Chen J. Y. Tu X. K. (2022). Research progress on postoperative transient neurological dysfunction in pediatric and adult patients with Moyamoya disease after revascularization surgery. Clin. Neurol. Neurosurg. 217:107254. doi: 10.1016/j.clineuro.2022.107254, PMID: 35512575

[ref6] Egashira Y. Yamauchi K. Enomoto Y. Nakayama N. Yoshimura S. Iwama T. (2017). Disruption of cortical arterial network is associated with the severity of transient neurologic events after direct bypass surgery in adult Moyamoya disease. World Neurosurg. 100, 311–315. doi: 10.1016/j.wneu.2017.01.039, PMID: 28109863

[ref7] Esposito G. Sebok M. Amin-Hanjani S. Regli L. (2018). Cerebral bypass surgery: level of evidence and grade of recommendation. Acta Neurochir. Suppl. 129, 73–77. doi: 10.1007/978-3-319-73739-3_10, PMID: 30171316

[ref8] Fahlström M. Lewén A. Enblad P. Larsson E. M. Wikström J. (2020). High intravascular signal arterial transit time artifacts have negligible effects on cerebral blood flow and cerebrovascular reserve capacity measurement using single postlabel delay arterial spin-labeling in patients with Moyamoya disease. AJNR Am. J. Neuroradiol. 41, 430–436. doi: 10.3174/ajnr.A6411, PMID: 32115416 PMC7077908

[ref9] Federau C. Christensen S. Zun Z. Park S. W. Ni W. Moseley M.. (2017). Cerebral blood flow, transit time, and apparent diffusion coefficient in Moyamoya disease before and after acetazolamide. Neuroradiology 59, 5–12. doi: 10.1007/s00234-016-1766-y, PMID: 27913820 PMC8006793

[ref10] Fujimura M. Inoue T. Shimizu H. Saito A. Mugikura S. Tominaga T. (2012). Efficacy of prophylactic blood pressure lowering according to a standardized postoperative management protocol to prevent symptomatic cerebral hyperperfusion after direct revascularization surgery for Moyamoya disease. Cerebrovasc. Dis. 33, 436–445. doi: 10.1159/000336765, PMID: 22456617

[ref11] Fujimura M. Mugikura S. Kaneta T. Shimizu H. Tominaga T. (2009). Incidence and risk factors for symptomatic cerebral hyperperfusion after superficial temporal artery-middle cerebral artery anastomosis in patients with Moyamoya disease. Surg. Neurol. 71, 442–447. doi: 10.1016/j.surneu.2008.02.031, PMID: 18514264

[ref12] Fujimura M. Shimizu H. Inoue T. Mugikura S. Saito A. Tominaga T. (2011). Significance of focal cerebral hyperperfusion as a cause of transient neurologic deterioration after extracranial-intracranial bypass for Moyamoya disease: comparative study with non-moyamoya patients using n-isopropyl-p-[(123)i]iodoamphetamine single-photon emission computed tomography. Neurosurgery 68:957–64; discussion 964-965. doi: 10.1227/NEU.0b013e318208f1da, PMID: 21221039

[ref13] Fukui M. (1997). Guidelines for the diagnosis and treatment of spontaneous occlusion of the circle of Willis (“moyamoya” disease). Research committee on spontaneous occlusion of the circle of Willis (Moyamoya disease) of the ministry of health and welfare, Japan. Clin. Neurol. Neurosurg. 99 Suppl 2, S238–S240. doi: 10.1016/S0303-8467(97)00082-6, PMID: 9409446

[ref14] Fung L. W. Thompson D. Ganesan V. (2005). Revascularisation surgery for paediatric moyamoya: a review of the literature. Childs Nerv. Syst. 21, 358–364. doi: 10.1007/s00381-004-1118-9 15696334

[ref15] Gao F. Zhao W. Zheng Y. Li S. Lin G. Ji M.. (2019). Phase-contrast magnetic resonance imaging analysis of cerebral hyperperfusion syndrome after surgery in adult patients with Moyamoya disease. World Neurosurg. 129, e48–e55. doi: 10.1016/j.wneu.2019.04.191, PMID: 31051310

[ref16] Goetti R. O’Gorman R. Khan N. Kellenberger C. J. Scheer I. (2013). Arterial spin labelling mri for assessment of cerebral perfusion in children with Moyamoya disease: comparison with dynamic susceptibility contrast mri. Neuroradiology 55, 639–647. doi: 10.1007/s00234-013-1155-8, PMID: 23404242

[ref17] Hayashi K. Horie N. Suyama K. Nagata I. (2012). Incidence and clinical features of symptomatic cerebral hyperperfusion syndrome after vascular reconstruction. World Neurosurg. 78, 447–454. doi: 10.1016/j.wneu.2011.10.041 22120558

[ref18] Hayashi T. Shirane R. Fujimura M. Tominaga T. (2010). Postoperative neurological deterioration in pediatric Moyamoya disease: watershed shift and hyperperfusion. J. Neurosurg. Pediatr. 6, 73–81. doi: 10.3171/2010.4.PEDS09478, PMID: 20593991

[ref19] Houkin K. Nakayama N. Kuroda S. Nonaka T. Shonai T. Yoshimoto T. (2005). Novel magnetic resonance angiography stage grading for Moyamoya disease. Cerebrovasc. Dis. 20, 347–354. doi: 10.1159/000087935, PMID: 16131804

[ref20] Huang W. Fang X. Li S. Mao R. Ye C. Liu W.. (2022). Shunt surgery efficacy is correlated with baseline cerebrum perfusion in idiopathic normal pressure hydrocephalus: a 3d pulsed arterial-spin labeling study. Front. Aging Neurosci. 14:797803. doi: 10.3389/fnagi.2022.797803, PMID: 35283746 PMC8906880

[ref21] Iwata T. Mori T. Miyazaki Y. Tanno Y. Kasakura S. Aoyagi Y. (2014). Global oxygen extraction fraction by blood sampling to anticipate cerebral hyperperfusion phenomenon after carotid artery stenting. Neurosurgery 75:546–51; discussion 551. doi: 10.1227/NEU.0000000000000485, PMID: 24991711

[ref22] Kazumata K. Ito M. Tokairin K. Ito Y. Houkin K. Nakayama N.. (2014). The frequency of postoperative stroke in Moyamoya disease following combined revascularization: a single-university series and systematic review. J. Neurosurg. 121, 432–440. doi: 10.3171/2014.1.JNS13946, PMID: 24605834

[ref23] Li Y. Wang A. R. Steinberg G. K. (2022). Safety and efficacy of induced hypertension and hypervolemia in preventing neurologic complications after combined direct and indirect bypass in hemorrhagic-onset Moyamoya disease. World Neurosurg. 160, e381–e387. doi: 10.1016/j.wneu.2022.01.017, PMID: 35026459

[ref24] Lu J. Zhao Y. Ma L. Chen Y. Li M. Chen X.. (2019). Predictors and clinical features of transient neurological events after combined bypass revascularization for Moyamoya disease. Clin. Neurol. Neurosurg. 186:105505. doi: 10.1016/j.clineuro.2019.105505, PMID: 31622898

[ref25] McDonald R. J. McDonald J. S. Kallmes D. F. Jentoft M. E. Murray D. L. Thielen K. R.. (2015). Intracranial gadolinium deposition after contrast-enhanced mr imaging. Radiology 275, 772–782. doi: 10.1148/radiol.15150025, PMID: 25742194

[ref26] Mukerji N. Cook D. J. Steinberg G. K. (2015). Is local hypoperfusion the reason for transient neurological deficits after sta-mca bypass for Moyamoya disease? J. Neurosurg. 122, 90–94. doi: 10.3171/2014.8.JNS132413, PMID: 25343178

[ref27] Noguchi T. Kawashima M. Nishihara M. Hirai T. Matsushima T. Irie H. (2013). Arterial spin-labeling mr imaging in Moyamoya disease compared with clinical assessments and other mr imaging findings. Eur. J. Radiol. 82, e840–e847. doi: 10.1016/j.ejrad.2013.08.040, PMID: 24055185

[ref28] Østergaard L. Engedal T. S. Aamand R. Mikkelsen R. Iversen N. K. Anzabi M.. (2014). Capillary transit time heterogeneity and flow-metabolism coupling after traumatic brain injury. J. Cereb. Blood Flow Metab. 34, 1585–1598. doi: 10.1038/jcbfm.2014.131 25052556 PMC4269727

[ref29] Park W. Park E. S. Lee S. Park J. C. Chung J. Lee J. M.. (2018). Intracranial hemorrhage after superficial temporal artery-middle cerebral artery direct anastomosis for adults with Moyamoya disease. World Neurosurg. 119, e774–e782. doi: 10.1016/j.wneu.2018.07.266, PMID: 30096496

[ref30] Research Committee on the Pathology and Treatment of Spontaneous Occlusion of the Circle of Willis, and Health Labour Sciences Research Grant for Research on Measures for Infractable Diseases (2012). Guidelines for diagnosis and treatment of moyamoya disease (spontaneous occlusion of the circle of Willis). Neurol. Med. Chir. 52, 245–266. doi: 10.2176/nmc.52.245, PMID: 22870528

[ref31] Scott R. M. Smith E. R. (2009). Moyamoya disease and Moyamoya syndrome. N. Engl. J. Med. 360, 1226–1237. doi: 10.1056/NEJMra0804622 19297575

[ref32] Shimada Y. Kojima D. Yoshida J. Kobayashi M. Yoshida K. Fujiwara S.. (2018). Transient symptomatic downregulation of cortical neurotransmitter receptor function due to cerebral hyperperfusion after arterial bypass surgery for a patient with ischemic Moyamoya disease. Neurol. Med. Chir. 58, 481–484. doi: 10.2176/nmc.cr.2018-0143, PMID: 30369534 PMC6236211

[ref33] Soman S. Dai W. Dong L. Hitchner E. Lee K. Baughman B. D.. (2020). Identifying cardiovascular risk factors that impact cerebrovascular reactivity: an asl mri study. J. Magn. Reson. Imaging 51, 734–747. doi: 10.1002/jmri.26862, PMID: 31294898 PMC6954347

[ref34] van Mook W. N. Rennenberg R. J. Schurink G. W. van Oostenbrugge R. J. Mess W. H. Hofman P. A.. (2005). Cerebral hyperperfusion syndrome. Lancet Neurol. 4, 877–888. doi: 10.1016/S1474-4422(05)70251-9 16297845

[ref35] Wang Z. Aguirre G. K. Rao H. Wang J. Fernández-Seara M. A. Childress A. R.. (2008). Empirical optimization of asl data analysis using an asl data processing toolbox: ASLtbx. Magn. Reson. Imaging 26, 261–269. doi: 10.1016/j.mri.2007.07.003, PMID: 17826940 PMC2268990

[ref36] Yun T. J. Paeng J. C. Sohn C. H. Kim J. E. Kang H. S. Yoon B. W.. (2016). Monitoring cerebrovascular reactivity through the use of arterial spin labeling in patients with Moyamoya disease. Radiology 278, 205–213. doi: 10.1148/radiol.2015141865, PMID: 26197057

[ref37] Zaharchuk G. Bammer R. Straka M. Shankaranarayan A. Alsop D. C. Fischbein N. J.. (2009). Arterial spin-label imaging in patients with normal bolus perfusion-weighted mr imaging findings: pilot identification of the borderzone sign. Radiology 252, 797–807. doi: 10.1148/radiol.2523082018, PMID: 19703858 PMC6939961

[ref38] Zaharchuk G. Do H. M. Marks M. P. Rosenberg J. Moseley M. E. Steinberg G. K. (2011). Arterial spin-labeling mri can identify the presence and intensity of collateral perfusion in patients with Moyamoya disease. Stroke 42, 2485–2491. doi: 10.1161/STROKEAHA.111.616466, PMID: 21799169 PMC3164217

[ref39] Zhang M. Tang J. Liu N. Xue Y. Ren X. Fu J. (2020). Postoperative functional outcomes and prognostic factors in two types of adult moyamoya diseases. J. Stroke Cerebrovasc. Dis. 29:104846. doi: 10.1016/j.jstrokecerebrovasdis.2020.104846, PMID: 32439351

